# The Cognitive Mechanisms of the Positivity Reactivity Effect on Word Recognition Memory

**DOI:** 10.3390/jintelligence14030047

**Published:** 2026-03-11

**Authors:** Baike Li, Chunliang Yang

**Affiliations:** 1 School of Psychology, Liaoning Normal University, Dalian 116029, China; baikeli@lnnu.edu.cn; 2 Institute of Developmental Psychology, Faculty of Psychology, Beijing Normal University, Beijing 100875, China; 3 Beijing Key Laboratory of Applied Experimental Psychology, National Demonstration Center for Experimental Psychology Education, Faculty of Psychology, Beijing Normal University, Beijing 100875, China

**Keywords:** judgments of learning, positive reactivity effect, word recognition memory, enhanced learning engagement

## Abstract

JOLs are widely used to measure metacognitive monitoring, yet their elicitation can reactively enhance memory—a phenomenon known as the positive reactivity effect. The enhanced engagement theory posits that JOLs improve memory by increasing attentional and cognitive engagement during encoding, but direct experimental evidence remains scarce. Across three experiments, we directly manipulated key components of learning engagement—attentional focus (via silent vs. aloud production), cognitive effort (via massed vs. spaced repetition), and motivational involvement (via standard vs. time-saving instructions)—while assessing their impact on the JOL reactivity effect in word recognition memory. Results consistently demonstrated robust positive reactivity effects, critically, the magnitude of these effects was significantly attenuated under high-engagement conditions (aloud reading, spaced learning, and heightened motivation). These converging findings provide the first direct, multi-method experimental support for the enhanced engagement theory, specifying that making JOLs benefit memory most when baseline engagement is low. The results delineate boundary conditions under which making JOLs yield beneficial effects and provide practical insights into leveraging JOLs to regulate engagement in real-world learning environments.

## 1. Introduction

Metacognition—specifically, the ability to monitor and regulate one’s cognitive processes—plays a central role in self-regulated learning (Bjork et al., 2013). A core component of metacognitive monitoring is the judgment of learning (JOL), through which learners predict their likelihood of remembering studied information on a future test (Dunlosky & Tauber, 2016; Metcalfe & Finn, 2013). Traditionally, JOLs have been viewed as passive reflections of an individual’s metamemory state, serving to guide subsequent study decisions about what, when, and how to learn (Rhodes & Tauber, 2011).

However, recent evidence challenges this static perspective by demonstrating that the act of making JOLs can itself reactively alter—and typically enhance—memory performance, a phenomenon termed the reactivity effect (e.g., Double et al., 2018; Li et al., 2023; Maxwell & Huff, 2024; Myers et al., 2025; Soderstrom et al., 2015). This finding indicates that JOLs are not merely diagnostic outputs of monitoring but can actively shape the encoding process, likely by directing attention, reallocating cognitive resources, or increasing the perceived importance of the material (Chang & Brainerd, 2024; Double et al., 2018; Myers et al., 2020). In word recognition tasks, for example, prompting item-by-item JOLs reliably enhances recognition accuracy for words that received JOLs compared to those studied without JOLs (Li et al., 2022, 2023).

The positive reactivity effect holds significant implications for theories concerning how metacognitive processes support learning and memory. Recent studies utilizing Chinese two-character words have demonstrated that making JOLs can boost recognition performance across different age groups, suggesting a potential role for JOL reactivity in cognitive development as well as adult learning (Li et al., 2022). A meta-analysis further indicates that, overall, making JOLs yields a small-to-moderate memory benefit, although the effect size varies substantially across studies and tasks (Double et al., 2018). This variability raises a pivotal theoretical question: Under what conditions does eliciting JOLs most strongly enhance learning, and what mechanisms underlie these benefits? Answering this question is essential for refining theories of metacognition and for optimizing the application of JOLs in educational contexts.

A large-sample study (N = 284) examined how individual differences in core cognitive abilities—working memory capacity (WMC), attentional control (AC), episodic memory (EM), and fluid intelligence (gF)—relate to the magnitude of the JOL reactivity effect (W. B. Zhao et al., 2025). WMC uniquely and positively predicted JOL reactivity, even after controlling for AC, EM, and gF. In contrast, after accounting for WMC, EM, and gF, AC showed a negative association with JOL reactivity. Together, these findings suggest that the memory benefit of making JOLs varies across individuals.

The variability in the JOL reactivity effect across individuals highlights the need to understand why the effect differs and how metacognitive strategies might be tailored to different learners (W. B. Zhao et al., 2025). However, individual-differences studies mainly provide correlational evidence. A necessary next step is to test proposed mechanisms experimentally by identifying the boundary conditions under which reactivity is most likely to occur. Doing so can explain why the effect varies across people and learning contexts and can move the field from description toward a clearer causal account of its cognitive basis. This approach also has practical value because it specifies when, and for whom, making JOLs is most beneficial.

One influential account is the enhanced engagement theory. It proposes that making JOLs increases learners’ attention and effort during encoding, which promotes deeper processing and improves later memory (Li et al., 2022, 2023). Eliciting a JOL may prompt learners to pause, reconsider the item, and reflect on how well they will remember it. This brief self-evaluation can reduce mind-wandering and encourage more elaborative or distinctive processing (Finn, 2008; Seli et al., 2016, 2019). Consistent with this view, the positive reactivity effect tends to be larger when baseline engagement is low—for example, when materials are monotonous, learners are prone to task-unrelated thoughts, or motivation is reduced (Li et al., 2022).

However, most evidence for the enhanced engagement theory remains indirect because engagement is usually inferred from outcomes rather than manipulated experimentally. Studies linking individual differences in cognitive resources (e.g., WMC and gF) to both metacognitive monitoring and effective strategy use suggest that the ability to mobilize cognitive resources relates to the magnitude of JOL reactivity (Jordão et al., 2019; Krawietz et al., 2012; Tauber & Witherby, 2019; W. B. Zhao et al., 2025). Importantly, several well-established encoding manipulations can directly increase engagement during study, offering a more decisive test of the theory. For example, reading words aloud rather than silently typically promotes deeper encoding (Bodner et al., 2014; Forrin et al., 2012; MacLeod & Bodner, 2017). Spaced, compared to massed, repetitions are also associated with more sustained attention and consolidation processes (Pastötter et al., 2011). Finally, instructions that emphasize efficiency or performance goals can increase motivational involvement during encoding (Kang & Pashler, 2014; Seli et al., 2016).

These findings suggest that attentional focus, cognitive effort, and motivational involvement shape both baseline learning and the size of the JOL reactivity effect. Accordingly, if eliciting JOLs benefit memory mainly by increasing engagement, their advantage should be largest when engagement is low (e.g., silent reading, massed learning, standard instructions) and smaller when engagement is already elevated by manipulations such as aloud reading, spaced repetitions, or motivationally enriched instructions (Li et al., 2022, 2023). To date, few studies have experimentally manipulated these distinct components of engagement within a single framework to test this moderation prediction.

The present study provides a direct test of the enhanced engagement theory by examining whether increasing engagement reduces the positive JOL reactivity effect in word recognition. Across three experiments, we compared recognition for words studied with versus without JOLs while manipulating three components of engagement: attentional engagement via the reading method (silent vs. aloud; Experiment 1), cognitive effort via spacing (massed vs. spaced; Experiment 2), and motivational engagement via instructional framing (control vs. motivation; Experiment 3). This approach tests whether the size of the reactivity effect depends on which component of engagement is increased, helping to clarify when and why eliciting JOLs is most likely to benefit memory.

## 2. Experiment 1

Experiment 1 probed into attentional engagement by means of a production-effect manipulation (aloud vs. silent reading), aiming to explore whether elevated engagement would weaken the positive reactivity effect of JOLs.

### 2.1. Methods

#### 2.1.1. Participants

A pilot study with ten participants was conducted to estimate the required sample size, following the identical procedure to the formal experiment. The results showed that the interaction effect size (*η*_p_^2^) between the learning method (JOL vs. No-JOL) and production method (silent vs. aloud) reached 0.27. A power analysis via G*Power 3.0 (Faul et al., 2007) indicated that a sample of approximately 32 participants was needed to detect a significant interaction (*α* = 0.05, power = 0.80). Ultimately, 34 healthy participants (*M* age = 23.08 years, *SD* = 3.29; 25 females) were recruited from the participant pool of Beijing Normal University (BNU). All participants provided written informed consent prior to the experiment, completed the task individually in a sound-attenuated cubicle, and received monetary compensation for their participation. The study protocol was approved by the Institutional Review Board of the Faculty of Psychology at BNU.

#### 2.1.2. Materials

The experimental stimuli comprised 352 two-character Chinese words selected from the SUBTLEX-CH Chinese word database (Cai & Brysbaert, 2010), with word frequencies ranging from 0.03 to 19.44 per million tokens. Among these, 32 words were used for practice trials, and the remaining 320 words were adopted for the formal experiment. In the formal experiment, 160 words were presented during the learning phase as “old” items in the subsequent recognition test, while the other 160 unstudied words served as “new” lures.

To eliminate potential item-selection biases, the 160 studied words were randomly divided into four lists (40 words per list). Two lists were assigned to the JOL condition, and the other two to the No-JOL condition. For each list, half of the words were randomly colored red and the other half blue. The presentation order of words, color assignment, and the sequence of list learning were all randomized by a computer program. All stimuli were displayed using the Psychtoolbox toolbox in MATLAB 2019b (Kleiner et al., 2007).

#### 2.1.3. Design and Procedure

Experiment 1 involved a 2 (learning method: JOL vs. No-JOL) × 2 (production method: silent vs. aloud) within-subject design, with both the learning method and production method serving as within-subject variables.

Before the formal experiment, participants were informed that they would study four lists of Chinese words for a subsequent memory test. For two of the lists, they were required to make item-by-item JOL to predict their memory performance for each word, while no such judgments were needed for the other two lists. For each list, blue words were to be read silently and red words aloud, with the color-to-reading-mode (silent or aloud) mapping counterbalanced across participants. Critical instructions emphasized that participants should maintain equal learning effort for all words, as all items would be included in the subsequent recognition test. A practice session with the same procedure as the formal experiment was administered to familiarize participants with the task requirements.

**Figure 1:**
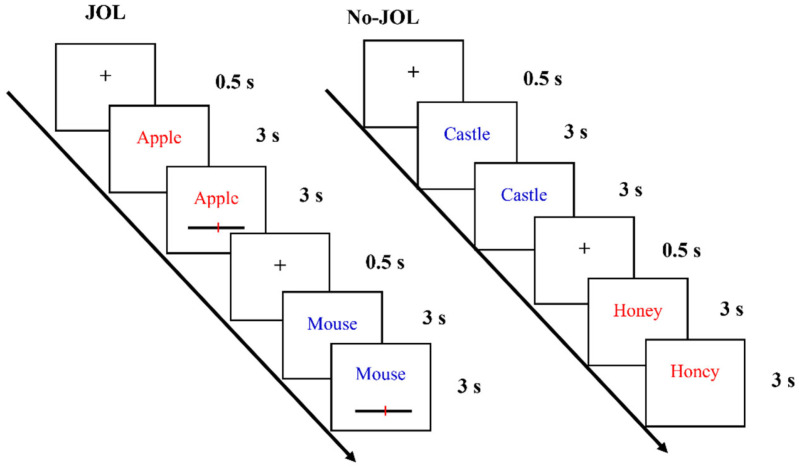
Flow illustration of the task procedure in Experiment 1.

The experimental procedure is illustrated in Figure 1. For the No-JOL condition, a fixation cross (+) was presented at the center of the screen for 0.5 s as the inter-stimulus interval, followed by a target word (blue or red) displayed for 6 s. Participants read red words aloud immediately and blue words silently, after which the next trial commenced. This cycle repeated until all words in the lists were studied.

For the JOL condition, the procedure was similar to the No-JOL condition with one key exception: each word was first presented for 3 s (during which participants read it silently or aloud as instructed), and then remained on the screen for an additional 3 s with a rating slider appearing below it. Participants were asked to use the slider to rate their likelihood of remembering the word in the subsequent test, on a scale from 0 (*absolutely certain not to remember*) to 100 (*absolutely certain to remember*). If a JOL was completed within the 3 s window, the word remained on the screen for the remaining time to ensure a total exposure duration of 6 s consistent with the No-JOL condition. If no judgment was made within the time limit, a reminder message appeared, prompting participants to complete the JOLs in a timely manner for subsequent trials; participants clicked the mouse to close the message and start the next trial.

Upon completion of the learning phase, participants performed a 5 min distractor task involving simple arithmetic calculations (e.g., 18 + 3 = ___). Subsequently, an old/new recognition test was administered, in which 160 studied words and 160 new lures were presented in a random order. Participants were asked to judge whether each word was “old” (studied) or “new” (unstudied) by pressing the “Z” key for old and “M” key for new, with no time limit and no feedback provided during the test.

### 2.2. Results and Discussion

The current analysis focused on the reactivity effect of JOLs, with item-by-item JOL ratings reported in Appendix A. No participants were excluded from the final analyses due to incomplete data or task noncompliance.

Recognition memory performance was quantified by discriminability *d*′, which reflects the ability to distinguish old, studied items from new lures and is calculated as the difference between the z-score of the hit rate (correct recognition of old items) and the z-score of the false-alarm rate (incorrect recognition of new items; Banks, 1970). Hit rates and false-alarm rates for all conditions are presented in Table A1 of Appendix B. A 2 × 2 repeated-measures analysis of variance (ANOVA) and paired-sample *t*-tests were conducted using JASP (Love et al., 2019). Bayes factors (*BF*_10_) were also reported to quantify the strength of evidence for the alternative hypothesis (*H*_1_) relative to the null hypothesis (*H*_0_), following Jeffreys’ evidence scale (Jeffreys, 1961): *BF*_10_ > 3 indicated moderate to strong evidence for *H*_1_, *BF*_10_ < 0.33 indicated moderate to strong evidence for *H*_0_, and values between 0.33 and 3 were considered anecdotal/inconclusive.

**Figure 2:**
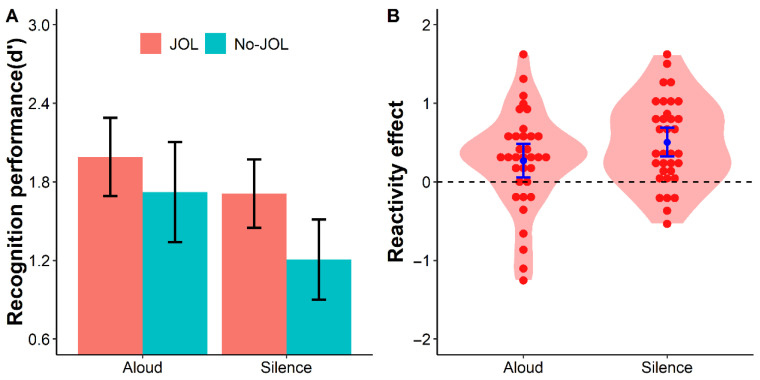
Recognition performance (**A**) and the JOL reactivity effect (**B**) in Experiment 1 as a function of condition. Violin plots depict the distributions of discriminability (*d*′; Panel **A**) and the reactivity effect (JOL − No-JOL; Panel **B**). Each red dot represents an individual participant, blue points indicate condition means, and error bars represent 95% confidence intervals. Key pattern: recognition performance is higher in the JOL than in the No-JOL condition, and the JOL reactivity effect is numerically smaller in the aloud condition than in the silent condition.

The repeated-measures ANOVA on *d*′ revealed a significant main effect of the production method, *F*(1, 33) = 38.51, *p* < .001, *η*_p_^2^ = 0.54, *BF*_10_ > 1000, indicating better recognition performance for words read aloud than silently. A significant main effect of the learning method was also found, *F*(1, 33) = 17.59, *p* < .001, *η*_p_^2^ = 0.35, *BF*_10_ = 146.93, showing that words in the JOL condition had higher *d*′ than those in the No-JOL condition. Most importantly, a significant interaction between the learning method and production method was observed, *F*(1, 33) = 7.99, *p* = .008, *η*_p_^2^ = 0.20, *BF*_10_ = 4.74 (see Figure 2).

Follow-up paired-sample *t*-tests were conducted to simple main effects of the learning method under each production condition. In the silent reading condition, *d*′ was significantly higher in the JOL condition (*M* = 1.71, *SD* = 0.78) than in the No-JOL condition (*M* = 1.21, *SD* = 0.91), with difference = 0.50, 95% CI [0.32, 0.69], *t*(33) = 5.41, *p* < .001, *d* = 0.93, *BF*_10_ > 1000. In the aloud reading condition, the JOL condition still yielded a significantly higher *d*′ (*M* = 1.99, *SD* = 0.89) than the No-JOL condition (*M* = 1.72, *SD* = 1.14), with difference = 0.27, 95% CI [0.05, 0.49], *t*(33) = 2.48, *p* = .02, *d* = 0.43, *BF*_10_ = 2.59.

A direct comparison of the JOL reactivity effect between the two production conditions showed that the reactivity effect was significantly larger in the silent condition (*M* = 0.50, *SD* = 0.54) than in the aloud condition (*M* = 0.27, *SD* = 0.63), difference = 0.24, 95% CI [0.07, 0.40], *t*(33) = 2.83, *p* = .008, *d* = 0.49, *BF*_10_ = 5.25.

These results confirm the robust positive reactivity effect of JOLs on word recognition memory (Li et al., 2022) and further demonstrate that manipulating attentional engagement via the production effect can modulate the magnitude of this effect. Specifically, the memory benefit of making JOLs is attenuated when learners’ attentional engagement is already elevated by aloud reading, which provides initial support for the enhanced engagement theory.

## 3. Experiment 2

Experiment 2 investigated the role of cognitive effort in the JOL reactivity effect by manipulating the presentation schedule of study items (massed vs. spaced repetition), with the aim of verifying whether increased cognitive effort during encoding would reduce the positive reactivity effect of JOLs.

### 3.1. Methods

#### 3.1.1. Participants

A pilot study with ten participants was conducted to determine the sample size, using the same procedure as the formal experiment. The results showed that the interaction effect size (*η*_p_^2^) between the learning method (JOL vs. No-JOL) and presentation schedule (massed vs. spaced) was 0.18. Power analysis via G*Power 3.0 (Faul et al., 2007) suggested that 38 participants were required to detect a significant interaction (*α* = 0.05, power = 0.80). To account for potential data loss, 42 participants (*M* age = 20.36 years, *SD* = 1.59; 38 females) were recruited from the BNU participant pool. All participants provided informed consent, completed the task individually in a soundproof cubicle, and received financial compensation. The study was approved by the Institutional Review Board of the Faculty of Psychology at BNU.

#### 3.1.2. Materials

Experiment 2 adopted a 2 (learning method: JOL vs. No-JOL) × 2 (presentation schedule: massed vs. spaced) within-subject design, with recognition memory *d*′ as the dependent variable. The experimental stimuli were 288 two-character Chinese words selected from the same database as Experiment 1, with word frequency matched to that in the first experiment.

Prior to the experiment, participants were informed that they would study a set of Chinese words, some of which would be presented once and others twice, and that a memory test would follow. The 288 words were randomly divided into four lists, with two lists assigned to the JOL condition and the other two to the No-JOL condition. For the JOL condition, participants were required to make item-by-item JOL for each word, while no JOLs were required for the No-JOL condition. Participants were instructed to learn all words with equal effort, as all items would be tested subsequently.

**Figure 3:**
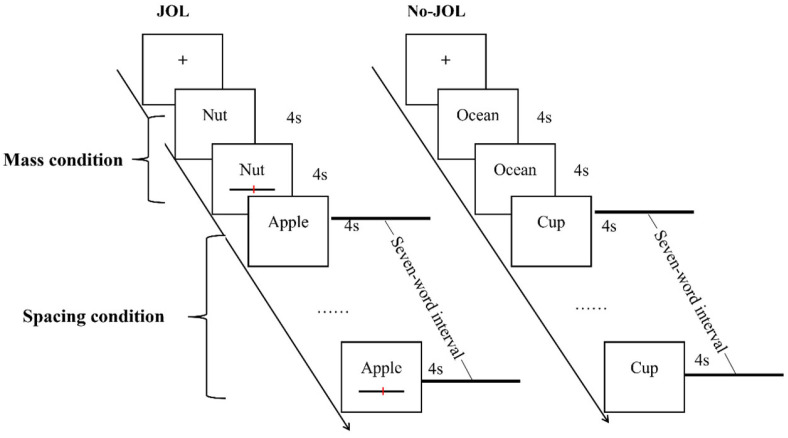
Flow illustration of the task procedure in Experiment 2.

The experimental procedure is illustrated in Figure 3. For No-JOL condition, a cross (“+”) appeared at the center of the screen for 0.5 s to mark the inter-stimulus interval. Immediately after, a word appeared on-screen for a total of 4 s. Then, the next trial started. This cycle repeated until the end of the list, with a new word studied in each cycle. For the JOL condition, the procedure was similar to the No-JOL condition except one difference. Specifically, when the word presented on the screen again, a slider would appear below it and participants were instructed to make a JOL using a scale ranging from 0 (*Sure I will not remember it*) to 100 (*Sure I will remember it*). After all words were learned, all participants were required to complete a 5 min distractor task and then complete an old/new recognition test.

### 3.2. Results and Discussion

No participants were excluded from the analyses due to task noncompliance or incomplete data. Recognition memory discriminability *d*′ was calculated as the primary index, with the independent variables being the learning method (JOL vs. No-JOL) and presentation schedule (massed vs. spaced). A 2 × 2 repeated-measures ANOVA and paired-sample *t*-tests were conducted using JASP, with Bayes factors reported to assess evidence strength for experimental effects.

**Figure 4:**
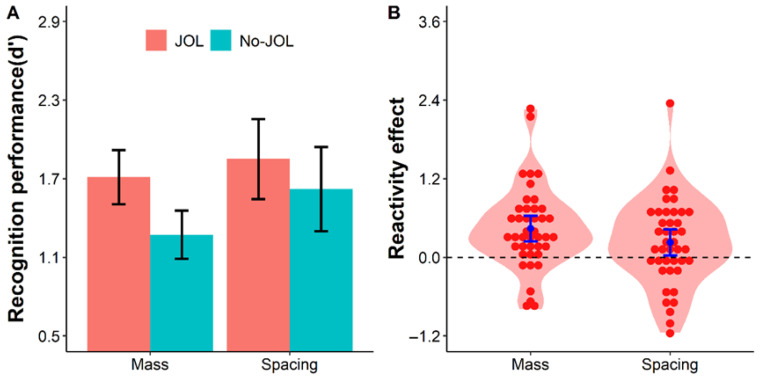
Recognition performance (**A**) and the JOL reactivity effect (**B**) in Experiment 2 as a function of condition. Violin plots depict the distributions of discriminability (*d*′; Panel **A**) and the reactivity effect (JOL − No-JOL; Panel **B**). Each red dot represents an individual participant, blue points indicate condition means, and error bars represent 95% confidence intervals. Key pattern: recognition performance is higher in the JOL than in the No-JOL condition, and the JOL reactivity effect is numerically smaller under the spacing condition than under the mass condition.

The repeated-measures ANOVA on *d*′ revealed a significant main effect of presentation schedule, *F*(1, 41) = 38.51, *p* = .018, *η*_p_^2^ = 0.13, *BF*_10_ = 3.21, indicating that spaced repetition led to better recognition performance than massed repetition. A significant main effect of the learning method was also found, *F*(1, 41) = 12.72, *p* < .001, *η*_p_^2^ = 0.24, *BF*_10_ = 35.82, showing that the JOL condition yielded higher discriminability than the No-JOL condition. A significant interaction between the learning method and presentation schedule was observed, *F*(1, 41) = 8.20, *p* = .007, *η*_p_^2^ = 0.17, *BF*_10_ = 4.84 (see Figure 4).

Follow-up paired-sample *t*-tests examined the simple main effect of the learning method under each presentation schedule. In the spaced repetition condition, *d*′ was significantly higher in the JOL condition (*M* = 1.85, *SD* = 1.01) than in the No-JOL condition (*M* = 1.62, *SD* = 1.06), difference = 0.23, 95% CI [0.02, 0.44], *t*(41) = 2.23, *p* = .032, *d* = 0.34, *BF*_10_ = 1.53. In the massed repetition condition, the JOL condition showed a substantially larger memory benefit, with *d*′ significantly higher (*M* = 1.71, *SD* = 0.68) than the No-JOL condition (*M* = 1.27, *SD* = 0.61), difference = 0.44, 95% CI [0.24, 0.64], *t*(41) = 4.47, *p* < .001, *d* = 0.69, *BF*_10_ = 376.52.

A direct comparison of the JOL reactivity effect between the two presentation schedules showed that the reactivity effect was significantly larger in the massed condition (*M* = 0.44, *SD* = 0.64) than in the spaced condition (*M* = 0.23, *SD* = 0.67), difference = 0.21, 95% CI [0.06, 0.36], *t*(41) = 2.86, *p* = .007, *d* = 0.44, *BF*_10_ = 5.75.

These results replicate the positive reactivity effect of JOLs in word recognition memory (Li et al., 2022) and extend the findings of Experiment 1 by demonstrating that cognitive effort (manipulated via presentation schedule) modulates the magnitude of this effect. Specifically, the memory benefit of making JOLs is significantly reduced when cognitive effort is already elevated by spaced repetition, which further supports the enhanced engagement theory.

## 4. Experiment 3

Experiment 3 examined the role of motivational engagement in the JOL reactivity effect via time-saving instructional framing to test whether increased learning motivation would attenuate the positive reactivity effect of JOLs on word recognition memory.

### 4.1. Methods

#### 4.1.1. Participants

A pilot study with 10 participants was conducted to estimate the required sample size, with the same procedure as the formal experiment. The results showed that the interaction effect size (*η*_p_^2^) between the learning method (JOL vs. No-JOL) and motivational group (control vs. motivation) was 0.15. Power analysis via G*Power 3.0 (Faul et al., 2007) indicated that at least 24 participants per group were needed to detect a significant interaction (*α* = 0.05, power = 0.80). Initially, 62 participants were recruited from the BNU participant pool; data from two participants were lost due to experimental procedure errors, resulting in a final sample of 60 participants (*M* age = 22.13 years, *SD* = 1.79; 54 females). Participants were randomly assigned to either the control group or the motivation group (30 participants per group), with group matching on age and gender. All participants provided written informed consent, completed the task individually in a sound-attenuated cubicle, and received monetary compensation. The study protocol was approved by the Institutional Review Board of the Faculty of Psychology at BNU.

#### 4.1.2. Material, Design, and Procedure

Experiment 3’s materials were identical to Experiment 1, and involved a 2 (learning method: JOL vs. No-JOL) × 2 (group: motivation vs. control) mixed design. The learning method acted as a within-subjects variable and the group as a between-subjects variable.

The procedure for the control group was similar to that of Experiment 1, except all words were presented in black font. Participants were informed they would study four-word lists for a later memory test, with 40 words in each list. They would make an item-by-item JOL for two lists (JOL condition), but not for the other two lists (No-JOL condition).

The procedure for the motivation group was very similar to that of the control group, with the only difference being that the motivation group was additionally informed before the experiment began: “*The entire learning task will last approximately 35 min. After learning all the word, you will participate in a memory test. If your test results do not reach the average level observed in previous experiments, you will need to spend an additional 35 min to complete the learning and testing tasks again. The learning and testing tasks will loop indefinitely until your final test score reaches or exceeds the average level.*” In contrast, the control group proceeded with the experiment as normal. After all words were learned, all participants were required to complete a 5 min distractor task and then complete a recognition test for old and new words.

To check whether the motivation manipulation was successful, following the test, both groups of participants were asked to honestly report how motivated they were to complete the learning task (i.e., *During the learning phase, how motivated are you to complete the task?*). The motivation levels were reported on a scale from 1 (*not motivated at all*) to 9 (*very motivated*). In addition, after the experiment, participants in the motivation group were further informed that they would not need to redo the task regardless of whether their test performance was above average. In summary, the only difference between the motivation group and the control group was that the motivation group received additional experimental instructions to increase their participation motivation.

### 4.2. Results and Discussion

No participants were excluded from the final analyses. First, the effectiveness of the motivational manipulation was verified via an independent-sample t-test on the subjective motivation ratings. The results showed that the motivation group reported a significantly higher level of learning motivation (*M* = 7.77, *SD* = 0.90) than the control group (*M* = 6.93, *SD* = 1.48), difference = 0.83, 95% CI [0.20, 1.47], *t*(58) = 2.63, *p* = .011, *d* = 0.68, *BF*_10_ = 4.41, confirming that the instructional framing successfully elevated participants’ motivational engagement during learning.

**Figure 5:**
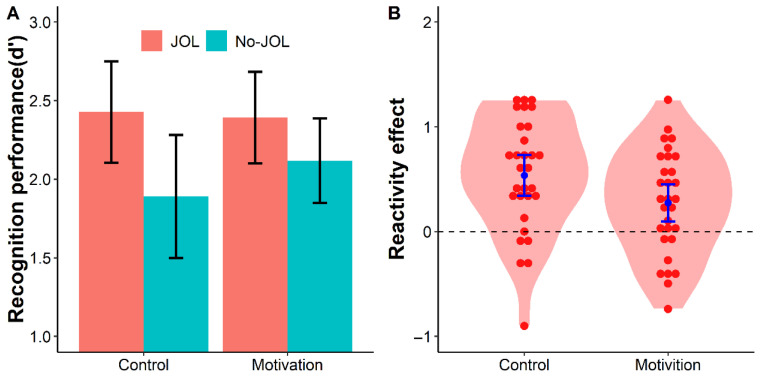
Recognition performance (**A**) and the JOL reactivity effect (**B**) in Experiment 3 as a function of group. Violin plots represent the distributions of *d*′ (**A**) and the reactivity effect (JOL − No-JOL; **B**). Red dots represent individual participants; blue points represent group means; error bars denote 95% confidence intervals. Key pattern: *d*′ is higher in the JOL than No-JOL condition in both groups, and the reactivity effect appears numerically smaller in the motivation group than in the control group.

Recognition memory discriminability *d*′ was analyzed using a 2 (learning method: JOL vs. No-JOL) × 2 (group: control vs. motivation) mixed ANOVA, with Bayes factors reported to assess effect strength. The analysis revealed a significant main effect of the learning method, *F*(1, 58) = 36.71, *p* < .001, *η*_p_^2^ = 0.39, *BF*_10_ > 1000, indicating that the JOL condition yielded significantly higher *d*′ than the No-JOL condition across both groups. The main effect of group was not significant, *F*(1, 58) = 0.19, *p* = .669, *η*_p_^2^ = 0.003, *BF*_10_ = 1.92, suggesting no overall difference in recognition performance between the control and motivation groups. A marginally significant interaction between the learning method and group was observed, *F*(1, 58) = 3.82, *p* = .055, *η*_p_^2^ = 0.06, *BF*_10_ = 1.17 (see Figure 5). The anecdotal evidence indicates there was not clear evidence favoring either *H*_1_ or *H*_0_.

Follow-up paired-sample *t*-tests examined the simple main effect of the learning method within each group. In the control group, *d*′ was significantly higher in the JOL condition (*M* = 2.43, *SD* = 0.90) than in the No-JOL condition (*M* = 1.89, *SD* = 1.10), difference = 0.54, 95% CI [0.33, 0.74], *t*(29) = 5.42, *p* < .001, *d* = 0.99, *BF*_10_ > 1000. In the motivation group, the JOL condition still showed a significant memory benefit, with *d*′ higher (*M* = 2.39, *SD* = 0.81) than the No-JOL condition (*M* = 2.12, *SD* = 0.75), difference = 0.28, 95% CI [0.09, 0.46], *t*(29) = 3.04, *p* = .005, *d* = 0.56, *BF*_10_ = 8.24.

Independent-sample *t*-tests compared recognition performance between the two groups under each learning method. Under the JOL condition, there was no significant difference in *d*′ between the motivation group (*M* = 2.39, *SD* = 0.81) and the control group (*M* = 2.43, *SD* = 0.90), difference = 0.04, 95% CI [−0.41, 0.48], *t*(58) = 0.16, *p* = .875, *d* = 0.04, *BF*_10_ = 0.27. Under the No-JOL condition, the motivation group showed a numerically higher *d*′ (*M* = 2.12, *SD* = 0.75) than the control group (*M* = 1.89, *SD* = 1.10), difference = 0.23, 95% CI [−0.26, 0.71], *t*(58) = 0.94, *p* = .354, *d* = 0.24, *BF*_10_ = 0.38, indicating inconclusive evidence regarding the group difference in reactivity.

A direct comparison of the JOL reactivity effect between the two groups was conducted via an independent-sample *t*-test. The results showed that the reactivity effect was numerically larger in the control group (*M* = 0.54, *SD* = 0.54) than in the motivation group (*M* = 0.28, *SD* = 0.49), difference = 0.26, 95% CI [−0.01, 0.53], *t*(58) = 1.95, *p* = 0.055, *d* = 0.51, *BF*_10_ = 1.28, with anecdotal evidence for a group difference.

These results confirm the persistent positive reactivity effect of JOLs even when motivational engagement is manipulated, and further suggest that elevated motivational engagement modulates the magnitude of the JOL reactivity effect. Specifically, the memory benefit of making JOLs is reduced in the high-motivation condition, which is consistent with the enhanced engagement theory and aligns with the findings of Experiments 1 and 2. Collectively, the three experiments demonstrate that the JOL reactivity effect is attenuated under high-engagement conditions—whether attentional, cognitive, or motivational—providing convergent experimental support for the core tenet of the enhanced engagement theory.

## 5. General Discussion

The present research aimed to clarify the cognitive mechanisms underlying the positive reactivity effect of judgments of learning (JOLs) on word recognition memory and to test the enhanced engagement theory using a rigorous, multi-method approach (for reviews, see Chang & Brainerd, 2024; Double et al., 2018; Li et al., 2023). Across three experiments, we manipulated engagement during encoding by targeting attentional engagement (Experiment 1), cognitive effort (Experiment 2), and motivational involvement (Experiment 3). A key contribution is that engagement was not only inferred from outcomes but was experimentally manipulated using well-established procedures from the production and spacing literatures (e.g., Bodner et al., 2014; Forrin et al., 2012; MacLeod & Bodner, 2017; Pastötter et al., 2011; Van Strien et al., 2007; X. Zhao et al., 2015). This approach allowed us to test whether the reactivity effect depends on baseline engagement. Across experiments, items that received JOLs showed better recognition than items studied without JOLs; however, the size of this benefit was smaller under higher-engagement conditions (aloud reading, spaced presentations, and motivational framing; (cf. Kang & Pashler, 2014; Li et al., 2022)). Together, these findings provide experimental evidence consistent with the enhanced engagement theory and identify boundary conditions under which eliciting JOLs is most likely to benefit memory.

### 5.1. Implications for Mechanism

Mechanistically, the reduced reactivity under higher-engagement conditions is consistent with the idea that making JOLs can enhance memory, in part, by increasing engagement during encoding (Li et al., 2022, 2023). When baseline engagement is relatively low, such as during silent reading or massed learning, prompting a JOL may encourage learners to pause and re-engage with the item. This pause can reduce shallow processing and produce a larger positive reactivity effect. In contrast, when encoding conditions already promote engagement, such as through distinctiveness from aloud reading or additional processing opportunities from spacing, the added benefit of a JOL appears smaller. This pattern fits a diminishing-returns interpretation: when attentional and elaborative resources are already strongly engaged by other manipulations, generating a JOL leaves less room to further improve encoding quality.

Our data may also clarify how the enhanced engagement account relates to alternative explanations for JOL reactivity, such as strategy-change accounts (Sahakyan et al., 2004). The larger reactivity effect observed when the baseline learning method is relatively less effective is consistent with the possibility that eliciting JOLs encourages learners to adopt more effective encoding strategies. At the same time, the presence of a positive reactivity effect even under higher-engagement conditions suggests that engagement-related and strategy-related mechanisms can operate together. One possibility is that generating a JOL supports memory through two complementary processes. It may increase effortful, focused processing, and it may also promote the adoption or refinement of encoding strategies based on ongoing metacognitive evaluation (Li et al., 2022). Because our experiments did not directly measure these processes, future work should aim to separate their contributions, for example by including process measures or manipulations that more selectively target engagement or strategy use (Chang & Brainerd, 2024; Dunlosky & Tauber, 2016).

### 5.2. Implications for Education

From an applied perspective, these findings highlight eliciting JOLs as a low-cost and flexible way to increase engagement during learning (Dunlosky & Tauber, 2016; Metcalfe & Finn, 2013; Mueller et al., 2013). The positive reactivity effect is strongest when baseline engagement is low, suggesting that JOLs may help compensate in everyday learning settings where attention and motivation often drop (Cox & Guthrie, 2001; Seli et al., 2016, 2019). When students study monotonous or highly familiar material, asking them to predict how well they will remember each item can shift learning from passive exposure to active monitoring. This shift can increase attention, effort, and depth of processing (Finn, 2008).

Practically, these findings support integrating structured JOL prompts into self-regulated learning routines and classroom instruction (Dunlosky & Tauber, 2016; Metcalfe & Finn, 2013). For example, educators can include brief JOL checks after segments of reading, vocabulary study, or concept learning, especially when learners are likely to mind-wander or disengage (Jordão et al., 2019; Seli et al., 2016, 2019). At the same time, our results suggest that JOLs work best as part of a broader set of engagement-supporting strategies rather than as a standalone technique. Combining JOLs with other effective encoding approaches, such as spacing, retrieval practice, and the production effect, may further improve both learning efficiency and long-term retention (Forrin et al., 2012; Kang & Pashler, 2014; Pastötter et al., 2011; X. Zhao et al., 2015).

### 5.3. Limitations and Future Research

Several limitations limit the generalizability of our conclusions and suggest clear directions for future work. First, we used only two-character Chinese words, and our participants were university students from a single cultural and educational context. In real educational settings, materials are often more complex, such as expository texts, diagrams, or multimodal content, and learners vary widely in age and cognitive ability. Future research should test whether the moderation of JOL reactivity by engagement generalizes to more complex materials, different task demands, such as free recall or comprehension, and more diverse samples, including children, older adults, and learners with attentional or executive difficulties (Jordão et al., 2019; Krawietz et al., 2012; Tauber & Witherby, 2019).

Second, although we increased engagement through production, spacing, and motivational instructions, we assessed engagement only indirectly using behavioral outcomes. We did not collect process-level measures, such as eye movements, physiological arousal, or neural indices of attention and elaboration. Future work can combine these behavioral manipulations with eye tracking, electrophysiology, or neuroimaging to more precisely characterize how eliciting JOLs shifts attention and resource allocation in real time and whether these changes mediate memory benefits (Allen et al., 2003; Mangels et al., 2001; Van Strien et al., 2007; Zhang et al., 2020; X. Zhao et al., 2015).

Third, our design does not fully distinguish the enhanced engagement account from strategy-change explanations. Although the attenuation of reactivity under higher-engagement conditions is consistent with an engagement-based view, learners may still adopt different encoding strategies when they are prompted to make JOLs (Begg & Snider, 1987; Bodner et al., 2014; Hourihan & MacLeod, 2008; Sahakyan et al., 2004). Future work can assess strategy use more directly using concurrent verbal protocols, post-task strategy questionnaires, or other process-tracing methods. Future studies can also examine how JOLs interact with mind-wandering and motivation during learning (Schmalz et al., 2023; Seli et al., 2016). Finally, experiments that selectively encourage or restrict specific strategies can help determine whether making JOLs benefit memory mainly by increasing engagement, by shifting strategies, or by influencing both processes together (Chang & Brainerd, 2024; Double et al., 2018; Dunlosky & Tauber, 2016).

## 6. Conclusions

The present study shows that the positive reactivity effect of JOL on recognition memory is reliable, but it tends to be smaller when learners are already highly engaged during encoding. This moderation pattern is consistent with the view that making JOLs benefits memory partly by increasing engagement, with the greatest gains emerging when baseline engagement is relatively low. More broadly, these findings help refine accounts of how metacognitive monitoring interacts with core cognitive processes and suggest that JOL prompts may be most beneficial when used alongside strategies that actively support learner engagement.

## Data Availability

Restrictions apply to the availability of these data. Data are available at https://osf.io/c8a7f (accessed on 8 November 2025).

## Appendix A. JOL Values and Relative Accuracy Results

### Appendix A.1. Experiment 1

In Experiment 1, the successful completion rate for JOL was 94.3% (*SD* = 6%). Regarding the average JOL values, the JOL value for the silent condition (*M* = 60.56, *SD* = 16.22) was significantly smaller than that for the aloud condition (*M* = 69.77, *SD* = 14.62), difference = −9.21, 95% CI [−13.89, −4.54], *t*(33) = −4.00, *p* < .001, *d* = −0.69, *BF*_10_ = 84.76. For each participant, the Gamma (G) correlation between JOL and memory performance was calculated as a measure of relative accuracy. Since two participants had a perfect accuracy rate of 1 in the aloud condition, their G could not be calculated. Excluding these two participants, a paired-sample t test found no significant difference in G between the aloud (*M* = 0.06, *SD* = 0.26) and the silent condition (*M* = 0.04, *SD* = 0.30), difference = 0.025, 95% CI [−13.89, −4.54], *t*(31) = 0.42, *p* = .68, *d* = 0.07, *BF*_10_ = 0.21.

### Appendix A.2. Experiment 2

In Experiment 2, the successful completion rate for JOL was 100% (*SD* = 1%). There was no significant difference in the average JOL values between the spacing condition (*M* = 60.39, *SD* = 13.22) and the mass condition (*M* = 58.88, *SD* = 13.77), difference = 0.80, 95% CI [−0.10, −3.12], *t*(33) = 1.90, *p* = .07, *d* = 0.29, *BF*_10_ = 0.85. For each participant, the Gamma (G) correlation was calculated as a measure of relative accuracy. Since one participant had a perfect accuracy rate of 1 in the spacing condition, their G could not be calculated. Excluding this participant, a paired-sample t test found no significant difference in G between the spacing condition (*M* = 0.14, *SD* = 0.32) and the mass condition (*M* = 0.15, *SD* = 0.36), difference = 0.011, 95% CI [−0.12, 14], *t*(31) = 0.18, *p* = .86, *d* = 0.03, *BF*_10_ = 0.17.

### Appendix A.3. Experiment 3

In Experiment 3, there was no significant difference in the successful completion rate of JOL between the control group (*M* = 99.0%, *SD* = 1%) and the motivation group (*M* = 99.1%, *SD* = 1%), difference = −1%, 95% CI [−0.5%, 0.5%], *t*(58) = −0.17, *p* = .87, *d* = −0.04, *BF*_10_ = 0.27. Regarding the average JOL values, there was no significant difference between the control group (*M* = 65.61, *SD* = 10.18) and the motivation group (*M* = 60.58, *SD* = 13.24), difference = 5.03, 95% CI [−1.08, 11.13], *t*(58) = 1.65, *p* = .11, *d* = −0.43, *BF*_10_ = 0.81. For each participant, the G was calculated as a measure of relative accuracy. The results of the paired-sample t test indicated a marginally significant difference in relative accuracy between the motivation group (*M* = 0.06, *SD* = 0.29) and the control group (*M* = 0.21, *SD* = 0.30), difference = 0.15, 95% CI [−0.1084 × 10^−4^, 0.31], *t*(58) = 2.00, *p* = .05, *d* = 0.52, *BF*_10_ = 1.37. The G for the motivation group did not significantly differ from 0, *t*(29) = 1.13, *p* = .27, *d* = 0.21, *BF*_10_ = 0.35. The G for the control group was significantly greater than 0, *t*(29) = 3.83, *p* < .001, *d* = 0.70, *BF*_10_ = 50.05.

## Appendix B. JOL Values and Relative Accuracy Results

**Table A1:** Hit rates and false-alarm rates (means and SDs) across experiments.

Experiment	Condition/Group	JOL Hit Rate (*M* ± *SD*)	No-JOL Hit Rate (*M* ± *SD*)	False Alarm (*M* ± *SD*)
Experiment 1	Aloud	0.82 ± 0.11	0.72 ± 0.18	0.20 ± 0.15
	Silence	0.75 ± 0.16	0.59 ± 0.19	
Experiment 2	Mass	0.80 ± 0.13	0.67 ± 0.17	0.24 ± 0.14
	Spacing	0.81 ± 0.13	0.74 ± 0.17	
Experiment 3	Control	0.86 ± 0.11	0.71 ± 0.21	0.14 ± 0.10
	Motivation	0.89 ± 0.11	0.83 ± 0.13	0.17 ± 0.11

*Note.* Values represent mean hit rates and false-alarm rates. False-alarm rates are calculated from responses to new items. *SD* = standard deviation.
